# What Happens in Between? Human Oscillatory Brain Activity Related to Crossmodal Spatial Cueing

**DOI:** 10.1371/journal.pone.0001467

**Published:** 2008-01-23

**Authors:** Maja U. Trenner, Hauke R. Heekeren, Markus Bauer, Konstanze Rössner, Rüdiger Wenzel, Arno Villringer, Manfred Fahle

**Affiliations:** 1 Berlin NeuroImaging Center, Neurologische Klinik und Poliklinik, Charité Universitätsmedizin Berlin, Berlin, Germany; 2 Max Planck Institute for Human Development, Berlin, Germany; 3 Centre for Cognitive Science, Human Neurobiology, University of Bremen, Bremen, Germany; Ecole Polytechnique Federale de Lausanne, Switzerland

## Abstract

Previous studies investigated the effects of crossmodal spatial attention by comparing the responses to validly versus invalidly cued target stimuli. Dynamics of cortical rhythms in the time interval between cue and target might contribute to cue effects on performance. Here, we studied the influence of spatial attention on ongoing oscillatory brain activity in the interval between cue and target onset. In a first experiment, subjects underwent periods of tactile stimulation (cue) followed by visual stimulation (target) in a spatial cueing task as well as tactile stimulation as a control. In a second experiment, cue validity was modified to be 50%, 75%, or else 25%, to separate effects of exogenous shifts of attention caused by tactile stimuli from that of endogenous shifts. Tactile stimuli produced: 1) a stronger lateralization of the sensorimotor beta-rhythm rebound (15–22 Hz) after tactile stimuli serving as cues versus not serving as cues; 2) a suppression of the occipital alpha-rhythm (7–13 Hz) appearing only in the cueing task (this suppression was stronger contralateral to the endogenously attended side and was predictive of behavioral success); 3) an increase of prefrontal gamma-activity (25–35 Hz) specifically in the cueing task. We measured cue-related modulations of cortical rhythms which may accompany crossmodal spatial attention, expectation or decision, and therefore contribute to cue validity effects. The clearly lateralized alpha suppression after tactile cues in our data indicates its dependence on endogenous rather than exogenous shifts of visuo-spatial attention following a cue independent of its modality.

## Introduction

Spatial cueing tasks have been widely used to investigate shifts of spatial attention [1], [2]. In these tasks, the position of a cue can be either congruent (valid) or incongruent (invalid) with the position of an upcoming target stimulus affecting the response to this target stimulus. Thus, the cue is not relevant to the specific task, but indicates where the target stimulus will likely appear. Such effects occur even when cue and target have different sensory modalities [e.g. 3]–[9].

Spatial attention comprises both, exogenous (externally-driven, bottom-up, involuntary, automatic) and endogenous (internally-driven, top-down, voluntary, non automatic) mechanisms. Whereas salient sensory events (e.g. a flash light) trigger transient automatic shifts of spatial attention, voluntarily shifted spatial attention can be held up for an extended time period [10]. The side initially advantaged by an exogenously induced attention shift is disadvantaged at stimulus-onset-asynchronies (SOAs) of more than about 400 ms, the so-called ‘inhibition of return’, IOR [11]–[14]. This phenomenon has been interpreted as an effect of either the reorientation of attention or of saccade preparation and inhibition [11], [15]–[17]. Several studies identified a largely overlapping cortical network involved in both exogenous and endogenous attention processes [18]–[22]. Mayer et al. (2004) used optimized stimulation designs for exogenous and endogenous spatial cueing, respectively, for example shorter SOAs for exogenous cueing [12]. They found a distinct network activated by voluntary attention shifts including the bilateral temporoparietal junction, bilateral superior temporal gyrus, right middle temporal gyrus, right frontal eye field, and left intraparietal sulcus. A recent study using event-related potentials (ERPs) compared exogenous attention shifts with endogenous attention shifts and a combination of both attention mechanisms [23]. In that study, exogenous attention tended to dominate at earlier stages of visual processing in the brain while endogenous attention tended to dominate at later stages. Correspondingly exogenous attention affected the late phase of the P1 ERP component whereas endogenous attention enhanced the P3 component. But the influences of both types of attention interfered with each other thereby providing evidence for partially separate but interacting attention systems.

Crossmodal spatial cueing has been extensively investigated by analyzing validity effects in ERPs. In these studies crossmodal attention shifts were either triggered by exogenous cues [e.g. 24], [25] or induced by endogenous cues [e.g. 26]–[28]. But spatial attention does not only influence brain activity that is phase-locked to the stimuli but also ongoing brain activity that is not phase-locked to the stimuli. In different experimental paradigms spatial attention enhanced high frequency oscillations in the gamma-band [29]–[34] (see [35], [36] for recent reviews) and modulated low frequency oscillations in the alpha-band [32], [37]–[44].

Behavioral and neural responses to the targets in a cueing task may depend on ongoing oscillatory brain activity during the time period preceding the target presentation. Only a few studies analyzed attention-related changes during spatial cueing induced by visual [39]–[41], [43] or auditory cues [44]. Mechanisms of crossmodal shifts of visuo-spatial attention following tactile cues remained so far uninvestigated.

Our study addresses two questions. First, we investigated how crossmodal shifts of visuo-spatial attention induced by tactile cues modulate cortical rhythms in the period between cue and target onset. The functional significance of these shifts was tested through their correlation with behavioral performance. Second, we aimed to dissociate effects of exogenous and endogenous attention. In a first experiment, we compared the effects of tactile cues in a crossmodal spatial cueing task with those of the identical tactile stimuli that did not serve as cues. This comparison allowed to distinguish between effects of top-down attention and response preparation processes on the one hand and effects of somatosensory stimulation and bottom-up attention processes on the other hand. To separate effects of exogenous shifts of attention by tactile stimuli from endogenous effects we conducted a second experiment, manipulating the validity of the cue to be either 50%, 75%, or 25%. In all three conditions, attention should first be automatically shifted to the side of the tactile cue–an exogenous attention shift. Hence, in the 75% condition the focus of endogenously shifted attention was congruent with the focus of exogenously triggered attention. On the other extreme, in the 25% condition endogenous attention should be shifted to the opposite side–since observers were aware of the fact that the cue appeared on the ‘wrong’ side. Thus, the focus of endogenous attention was incongruent with the focus of exogenous attention (endogenous attention shift). Effects depending on endogenous rather than exogenous attention should change their laterality in the 25% condition for identical stimuli. Therefore, this design allows to separate effects of exogenous shifts of attention by tactile stimuli from endogenous effects.

## Methods

### Subjects

Thirty-two subjects were paid to participate in one of the experiments:

*Experiment I:* Sixteen (8 female), mean age 25 years, range 21–35 years, right-handed (mean handedness score +82, range +60 to +100); *Experiment II:* 16 (12 female), mean age 27 years, range 20–31 years, right handed (mean handedness score +88, range +60 to +100).

None of the subjects had a history of neurological or psychiatric dysfunction. All participants reported normal or corrected-to-be-normal visual acuity and were right handed according to the Edinburgh Handedness Inventory [45]. Five additional participants were excluded because of too high impedances during electroencephalography (EEG) acquisition. The study was approved by the local ethics committee. Written and informed consent was obtained from each participant prior to investigation according to the declaration of Helsinki.

### Stimuli and Experimental Design

For tactile stimulation, we used two custom-made piezoceramic vibrators (PL127.11, dimensions 31.0 mm×9.6 mm×0.65 mm; PI Ceramic GmbH, Lederhose, Germany) driven by a home-made stimulator (Universitätsmedizin Charité, medical–technical laboratories, Berlin, Germany).

#### Experiment I

Each subject underwent three periods of stimulation in complete darkness in the following sequence: I. control task (named ‘control task pre’), II. crossmodal cueing task, III. control task post (named ‘control task post’). In each task 256 trials were presented. During the whole trial, subjects fixated a cross in the center of the screen.

##### Control task

In each trial a short vibro-tactile 60 Hz stimulus S_1_ (100 ms) with an average amplitude of 383 µm, was delivered randomly to the tip of either the right or the left index finger. Both fingers rested in a central position in front of the subject who was loosely holding the stimulators. To decrease effects of expectancy the length of the inter-trial-interval was jittered (in steps of 250 ms between 2000–3000 ms, mean 2500 ms).

##### Crossmodal cueing task

**Figure 1:**
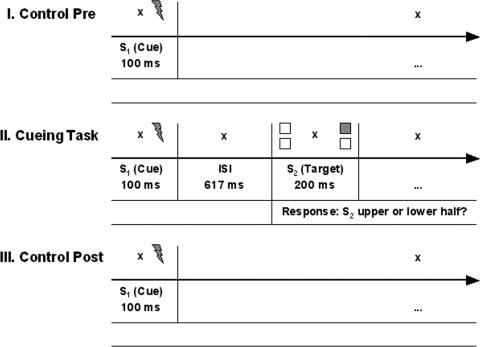
Experimental tasks. I. and III. Control task before and after the crossmodal spatial cueing task. Brief (100 ms) vibro-tactile S_1_ stimuli were randomly delivered to the left or right index finger. II. Time course of a valid experimental trial in the crossmodal spatial cueing task. Each trial began with a 100 ms vibro-tactile S_1_ stimulus delivered randomly to the left or right index finger. After an ISI of 617 ms a bright square was presented in one of the screen's quadrants for 200 ms. Subjects pressed the left or right foot pedal to indicate whether the square appeared in the upper or lower half of the screen. Three quarters of the S_2_ stimuli appeared on the same side as the S_1_ stimulus. During and in between trials-i.e. permanently-subjects fixated a cross in the center of the screen.

Trial number and parameters of stimulus S_1_ were identical to those in the control task. S_1 _was followed by a visual stimulus S_2_ (200 ms) at a stimulus onset asynchrony of 717 ms corresponding to an ISI of 617 ms (figure 1). S_2_, a white square (height 6.4°×width 0.9°), was presented randomly in one of the four quadrants of the screen (at a distance of 21.5° in horizontal and 13.3° in vertical direction from the central fixation point) on a TFT-monitor in front of the subject at a viewing distance of 40 cm. Observers were asked not to saccade to the target stimuli. During the practice phase, subjects were trained until they were able not to move their eyes towards the stimuli but to keep central fixation during lateralized visual stimulation. As in the control task, the inter-trial-interval was jittered. Subjects' task was to decide as fast and accurately as possible whether the visual stimulus appeared in the upper or lower hemifield; thus, S_1_s did not predict which foot to respond with.

Responses to target stimuli were given via foot pedal presses. Too early responses (earlier than 200 ms after target onset) were indicated by a brief 500 Hz feedback tone. The response scheme (right versus left foot pedal response to a stimulus in the upper versus lower half of the screen) was counter balanced across subjects. Feedback regarding mean reaction times and error rates was provided on the screen at the end of each block. In experiment I, S_2_ appeared on the same side as S_1 _in 75% of the trials (valid) and on the other side in 25% of the trials (invalid). Figure 1 depicts the time course of an experimental trial. Please note that in this study we focus on the period between the cue and the target stimulus (ISI), which is not influenced by the overt response to the targets.

#### Experiment II

Within each session, each subject underwent three experimental blocks of the crossmodal spatial cueing task in complete darkness (see cueing task in experiment I and in figure I) with 256 trials each. Depending on the validity proportion in the individual block, either 50%, 75% or 25% of the trials were valid. Subjects were informed about cue validity probability in advance and were asked to use this information to maximize response speed and accuracy. The session began with the 50% validity condition, then half of the subjects performed the 75% validity condition before the 25% cue validity condition while the other half experienced the 25% cue validity condition before the 75% validity condition.

### Data Acquisition of Behavioral Responses

Responses were scored as correct if the correct foot pedal was pressed within a time window lasting from 200 to 1300 ms after target onset. Errors of omission (no pedal press) and of commission (wrong pedal) were recorded separately. Mean reaction times were calculated for correct responses only.

### Data Acquisition EEG

The EEG was recorded with an amplifier (Brain Products GmbH, Munich, Germany) connected to Ag/AgCl electrodes (30 in *experiment I*, 42 in *experiment II*) mounted in an electrode cap (Easycap GmbH, Herrsching-Breitbrunn, Germany). Scalp positions were, in *experiment I*: F_z_, C_z_, P_z_, O_z_, Fp_1_, Fp_2_, F_3_, F_4_, FC_5_, FC_6_, FC_1_, FC_2_, C_3_, C_4_, F_7_, F_8_, T_7_, T_8_, CP_5_, CP_6_, P_3_, P_4_, P_7_, P_8_, O_1_, O_2_, PO_9_, PO_10_, TP_9_ and TP_10_; in *experiment II*: F_z_, C_z_, P_z_, O_z_, Fp_1_, Fp_2_, F_3_, F_4_, FT_7_, FT_8_, FC_5_, FC_6_, FC_3_, FC_4_, FC_1_, FC_2_, C_5_, C_6_, C_3_, C_4_, C_1_, C_2_, T_7_, T_8_, CP_5_, CP_6_, CP_3_, CP_4_, CP_1_, CP_2_, P_3_, P_4_, P_7_, P_8_, PO_3_, PO_4_, O_1_, O_2_, PO_9_, PO_10_, TP_9_ and TP_10_. The positions TP_9_ and TP_10_ refer to inferior temporal locations over the left and right mastoids, respectively. The TP_10_ electrode served as initial common reference, and a forehead electrode (AF_z_) served as the ground. All impedances were kept below 10 kΩ and were typically below 5 kΩ. The vertical electrooculogram (EOG) was monitored using two electrodes above and below the right eye, respectively, the horizontal EOG was recorded from two electrodes at the outer canthi of the eyes. All signals were filtered using a band-pass filter from 0.53 Hz to 70 Hz in *experiment I* and to 120 Hz in *experiment II* (12 dB/octave) and sampled at a rate of 500 Hz.

### Data Analysis EEG

Using Brain Vision Analyzer Software (Brain Products GmbH, Munich, Germany), continuous data were re-referenced to linked mastoids and segmented into epochs around the vibro-tactile stimulus: −720 ms to 1410 ms (control task); −720 ms to 1710 ms (cueing task, *experiment I*); −750 ms to 1610 ms (cueing task, *experiment II*). Automatic correction of vertical eye movements [46] and an artifact detection algorithm were run for an initial sorting of trials. All trials were then visually inspected for remaining artifacts. Trials with artifacts (including horizontal eye movements in the ISI) and trials with incorrect behavioral responses were discarded.

Time-frequency analysis was applied for all remaining trials. The event-related spectral power modulation of the cortical rhythms was analyzed using the EEGLAB v5.02 toolbox [47] under MATLAB v7.04 (Mathworks, Natick, MA). Trial-by-trial event-related spectral power changes were analyzed by the event-related spectral perturbation *(ERSP)* index [48]:
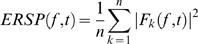
For *n* trials, *ERSP* gives the mean *F_k_(f,t)*, which is the spectral estimate of trial k at frequency f and time t. *F_k_(f,t)* was computed using a hanning-tapered sinusoidal wavelet (short-time DFT) transform with the number of cycles linearly rising from a minimum of 2 cycles for 3.9 Hz to a maximum of 25 cycles for 49.8 Hz within one analysis window. Individual subject's ERSP results were baseline-normalized by subtracting the mean baseline log power spectrum from each spectral estimate and thresholded by applying a significance threshold of p<0.05. To avoid any probability distribution assumption bootstrap statistics were applied comparing the data distributions against bootstrap distributions, which had been drawn at random from the pre-stimulus baseline and applied 200 times.

Post-hoc and additional time-frequency analysis was run for one time-frequency window (25–35 Hz, 600–700 ms) using the multi-taper method which offers optimal spectral concentration over the frequency range of interest (Fieldtrip toolbox, F.C. Donders Centre for Cognitive Neuroimaging, Nijmegen, Netherlands; http://www2.ru.nl/fcdonders/fieldtrip). The 25–35 Hz frequency band was analyzed with a window length of 400 ms and a spectral concentration of 2.5 Hz; the 700 ms preceding the cue stimulus served as baseline.

Time-frequency windows were defined to measure suppression of occipital alpha- and rolandic mµ-rhythm (7–13 Hz), suppression and rebound of central beta-rhythm (15–22 Hz) as well as gamma-rhythm enhancement (25–95 Hz) in the inter-stimulus-interval (ISI) between S_1_ and S_2_. The individual mean values for each time-frequency (T-F) window were extracted from each channel and regions of interest (ROIs) were defined.

*Experiment I:* Eleven T-F windows within the following time and frequency ranges were analyzed: 500–700 ms (7–13 Hz); 300–700 ms (15–22 Hz), 300–700 ms (25–45 Hz). ROIs: central (left: C_3_/CP_5_, right: C_4_/CP_6_), occipital (left: P_7_/O_1_/PO_9_, right: P_8_/O_2_/PO_10_) and prefrontal (left: Fp_1_, right: Fp_2_). *Experiment II:* Thirty-four T-F windows within the following time and frequency ranges were analyzed: 400–700 ms (8–12 Hz); 300–700 (15–22 Hz); 300–700 ms (25–95 Hz). ROIs: central (left: C_3_/CP_5_, right: C_4_/CP_6_), lateral occipital (left: P_7_/PO_3_, right: P_8_/PO_4_) and prefrontal (left: Fp_1_, right: Fp_2_).

First, event-related synchronization (ERS) or desynchronization (ERD) was tested for each combination of condition, ROI and T-F window (separately for the side contralateral and ipsilateral to the S_1_ stimulus). Second, in the case of significant ERS or ERD (one-tailed t-test) in at least one task, analyses of variance (ANOVAs) with repeated measures were conducted on the three-level factor TASK CONDITION (control pre, control post, cueing), the two-level factor ELECTRODE HEMISPHERE (right or left hemisphere) and the two-level factor S_1_ STIMULUS SIDE (right or left index finger). In *experiment II,* the factor TASK CONDITION was replaced by a three-level factor VALIDITY CONDITION (50%, 75%, 25%).

## Results

### Experiment I

#### Behavioral data

An ANOVA with repeated measures on the two-level factor VALIDITY (valid or invalid), the two-level factor S_1_ STIMULUS SIDE (right or left index finger), and the two-level factor S_2_ stimulus HEIGHT (upper or lower screen half) was performed on both, error rates and reaction times.

##### Errors

Errors were errors of commission (wrong key response) or omission (no or too fast key response). The percentage of errors was very low (valid: *Mean* = 3.4%, *SD* = 1.6; invalid: *Mean* = 5.1%, *SD* = 3.5) and did not exceed 5.0% in all but one condition, in which the error rate was 7.0%. Subjects made fewer errors in validly cued trials (VALIDITY, *F*(1,15) = 5.32, *p*<.04).

##### Reaction times (RTs)

RTs were faster in validly cued trials (valid: *Mean* = 538 ms, *SD* = 73.5; invalid: *Mean* = 559 ms, *SD* = 75.1) (VALIDITY, *F*(1,15) = 15.01, *p*<.001). Responses were also faster to S_2_s appearing in the lower half of the screen (HEIGHT, *F*(1,15) = 4.38, *p* = .05).

#### Time-frequency EEG

##### Occipital alpha-rhythm suppression (7–13 Hz)

**Figure 2:**
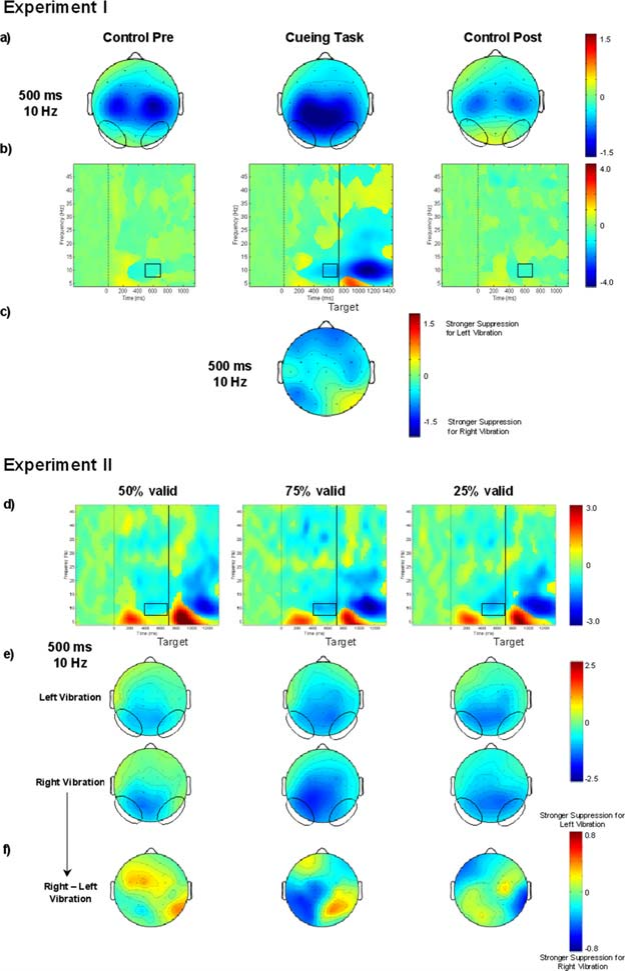
Grand average of occipital time-frequency modulations in experiments I+II. a)+e) 2-D topographies of the alpha-rhythm suppression in the ISI (500 ms, 10 Hz) are plotted. Ellipses enclose the occipital regions of interest. b+d) Grand averages of time-frequency matrices for the occipital regions of interest for the cueing task and the control tasks (b) separately for the three validity conditions (d). Zero on the x-axis corresponds to the onset of the S_1_ stimulus. The black vertical line in the results of the cueing task marks the onset of the S_2_ stimulus. Black squares designate the time-frequency window in which the alpha-rhythm suppression was analyzed. c+f) The topography plots illustrate the laterality of the alpha-rhythm suppression in the cueing task. The amount of stimulus-locked oscillations at various times and frequencies relative to the pre-stimulus baseline is color-coded and is expressed in *db*. In a), b), d), and e) positive values indicate an increased, while negative values indicate a decreased amount of stimulus-locked oscillations. In c) and f) positive values indicate stronger alpha-rhythm suppression after left than after right finger stimulation, while negative values indicate stronger alpha-rhythm suppression after right than left right finger stimulation.

Only in the cueing task a significant suppression of occipital alpha-rhythm occurred (figure 2a and b). It began about 300 ms after the S_1_ stimulus, increased after the S_2_ stimulus, and still continued 500 ms after the S_2_ stimulus (figure 2b, middle plot). In the ISI (500–700 ms). Suppression was significant both contra- and ipsilaterally to the attended side but stronger at contralateral sites (STIMULUS SIDE×ELECTRODE HEMISPHERE, F(1,15) = 6.79, p<0.02) (figure 2c).

##### Central mµ-rhythm suppression (7–13 Hz)

**Figure 3:**
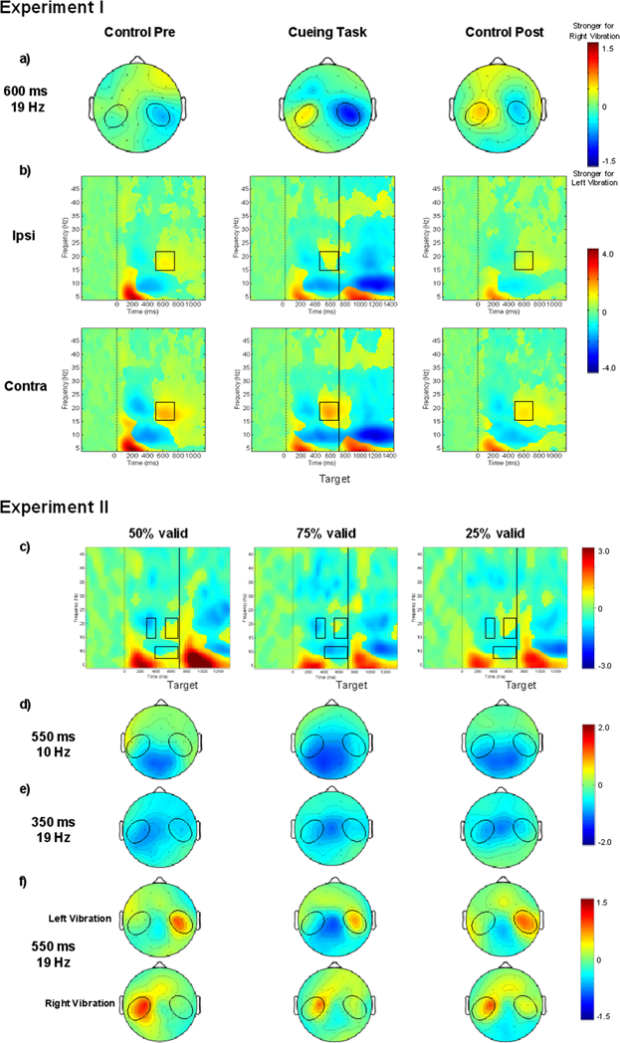
Grand average of rolandic time-frequency modulations in experiments I+II. a) 2-D topographies of the beta-rhythm-rebound in the ISI (600 ms, 19 Hz) are plotted. Ellipses enclose the central regions of interest. Plots illustrate the laterality of the beta-rhythm rebound. b)+c) Grand averages of time-frequency matrices for the central region of interest for the cueing task and the control tasks separately for the three validity conditions. Zero on the x-axis corresponds to the onset of the S_1_ stimulus. The black vertical line in the results of the cueing task marks the onset of the S_2_ stimulus. The black squares designate the time-frequency windows in which the suppression of the mµ-rhythm as well as the suppression and rebound of the beta-rhythm were seen. d-f) 2-D topographies for mµ-rhythm suppression (550 ms, 10 Hz) (d) and the beta-rhythm suppression (350 ms, 19 Hz) (e) are plotted. The beta-rhythm rebound (550 ms, 19 ms) (f) is shown separately for vibration stimuli to the right or to the left index finger. Ellipses in each topography plot enclose the central regions of interest. The amount of stimulus-locked oscillations at various times and frequencies relative to the pre-stimulus baseline is color coded and is expressed in *db*. In a) positive values indicate a stronger beta-rhythm after right than after left finger stimulation, while negative values indicate a stronger beta-rhythm after left than right finger stimulation. In b)- f) positive values indicate an increased, while negative values indicate a decreased amount of stimulus-locked oscillations.

In the cueing task the central mµ-rhythm was suppressed beginning about 300 ms after the S_1_ stimulus, getting stronger after the S_2_ stimulus and still continuing 500 ms after the S_2_. In the control tasks on the other hand, suppression was limited to the time range of 300–700 ms after the S_1_ (figure 3b).

##### Central beta-rhythm suppression & rebound (15–22 Hz)

The central beta-rhythm was suppressed in all three tasks in the period about 200–500 ms after the S_1_ stimulus (figure 3b). In the time window from 300 to 500 ms the suppression was stronger contralaterally (STIMULUS SIDE×ELECTRODE HEMISPHERE, F(1,15) = 5.52, p<0.03). In all tasks a beta rebound was observed within the 500–800 ms time range (figure 3b). In the 500–700 ms time window it was stronger contralaterally (STIMULUS SIDE×ELECTRODE HEMISPHERE, F(1,15) = 12.70, p<0.003) (figure 3a). This laterality differed between tasks; it was stronger in the cueing task than in the control task pre (TASK CONDITION×STIMULUS SIDE×ELECTRODE HEMISPHERE, F(1,15) = 3.50, p<0.04) (figure 3a) (see table 1).

**Table 1:** EEG results sensorimotor beta-rhythm rebound experiment I

Condition	Sensorimotor beta-rhythm rebound in *db*	
	Mean	Std. error
Contralateral Control_pre	.60	.27
Ipsilateral Control_pre	.36	.19
Contralateral Cueing task	.73	.28
Ipsilateral Cueing task	.11	.17
Contralateral Control_post	.60	.17
Ipsilateral Control_post	.27	.09

For the three experimental conditions (Control tasks, Cueing task), the mean sensorimotor beta-rhythm rebound (including standard error of the mean) in the time window from 500–700 ms is given.

##### Prefrontal gamma-rhythm enhancement (25–35 Hz)

**Figure 4:**
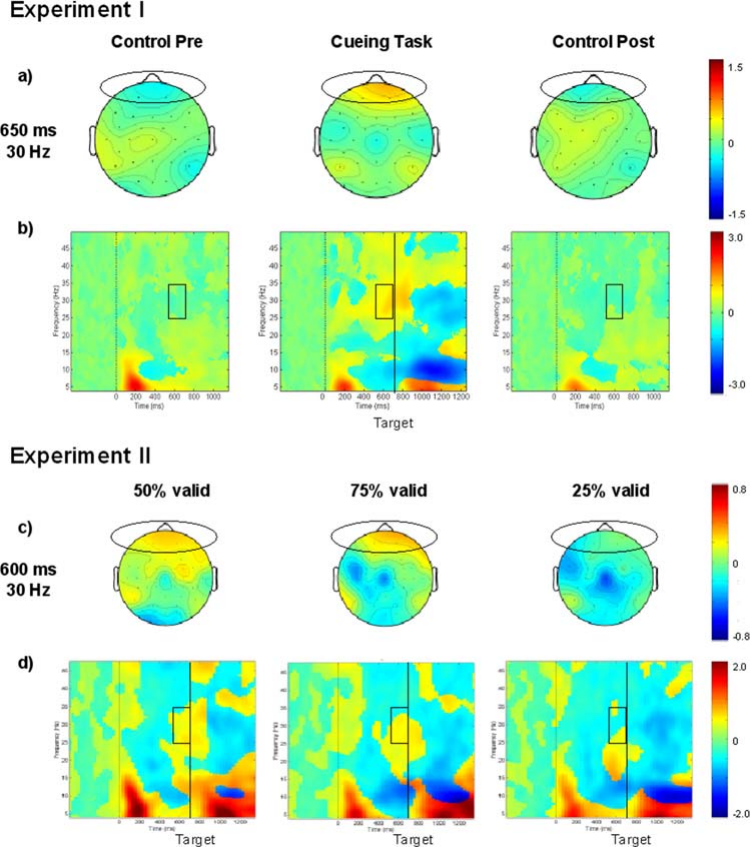
Grand average of prefrontal time-frequency modulations in experiments I+II. a)+c) 2-D topographies of the gamma-rhythm enhancement in the ISI between cue and target are plotted. Ellipses enclose the prefrontal regions of interest. b)+d) Grand averages of time-frequency matrices for the prefrontal region of interest for the cueing task and the control tasks (b) separately for the three validity conditions (d). Zero on the x-axis corresponds to the onset of the S_1_ stimulus. The black vertical line in the results of the cueing task marks the onset of the S_2_ stimulus. Black squares designate the time-frequency windows in which the gamma-enhancement was observed in the cueing task. The amount of stimulus-locked oscillations at various times and frequencies relative to the pre-stimulus baseline is color-coded and is expressed in *db*. Positive values indicate an increased, while negative values indicate a decreased amount of stimulus-locked oscillations.

Gamma-activity at prefrontal sites increased specifically in the cueing task immediately preceding the target presentation (600–700 ms) (TASK CONDITION F(1,15) = 4.73, p<0.02) (figure 4a, b). Although the prefrontal electrodes (Fp positions) and the upper eye electrodes are spaced closely to each other, the topography of the gamma-enhancement measured with these electrodes differed (see figure S1). In the ISI gamma-activity was stronger at prefrontal sites, while gamma-activity following the target presentation was more pronounced at the eye electrode.

#### Main EEG results-experiment I

Three results were observed for tactile cues in the cueing task but not for identical tactile stimuli in the control task: First, the occipital alpha-rhythm was suppressed; this suppression was stronger contralaterally to the stimulus. Second, the sensorimotor beta rebound was more strongly lateralized. Third, prefrontal gamma-activity increased.

### Experiment II

#### Behavioral data

Error rates and RTs were analyzed analogously to experiment I. Instead of the factor TASK CONDITION in experiment I the ANOVA involved a three-level factor VALIDITY CONDITION (50%, 75%, 25% validity). (See table 2 for an overview of the descriptive data.)

**Table 2:** Behavioral data experiment II

Condition	Reaction Time in ms		Error Rate in %	
	Mean	SD	Mean	SD
Valid_50	512.25	55.16	3.76	3.47
Invalid_50	518.14	59.88	3.81	3.10
Valid_75	512.07	63.91	3.68	3.01
Invalid_75	543.87	68.20	4.69	3.33
Valid_25	543.96	80.83	4.39	3.43
Invalid_25	522.28	72.06	4.43	3.26

For the three experimental conditions (50%, 75% or 25% cue validity), the mean reaction times and error rates are indicated with their standard deviations (SD). Responses to S_2_ stimuli could be either validly (valid) or invalidly (invalid) cued by the S_1_ stimulus. The S_2_ stimulus appeared at one of four possible positions on the screen.

##### Errors

The percentage of errors was very low (50% validity-valid: *M* = 3.8%, invalid: M = 3.8%; 75% validity-valid: *M* = 3.7%, invalid: M = 4.7%; 25% cue validity-valid: *M* = 4.4%, invalid: M = 4.4%). Errors did not exceed 6.0% in all but one sub- condition, in which the error rate was 6.3%.

##### Reaction times (RTs)

RTs were slightly faster in the 50% than in the 25% validity condition (VALIDITY CONDITION, *F*(1,15) = 3.51, *p*<.04; Bonferroni corrected post-hoc test, *p*<.05). To clarify the difference between validity effects in the three conditions (VALIDITY CONDITION×VALIDITY, *F*(1,15) = 19.3, *p*<.001) three separate ANOVAs were calculated for each validity condition. RTs were faster in validly cued trials in the 75% condition (VALIDITY, *F*(1,15) = 19.59, *p*<.001). In contrast, in the 25% validity condition RTs were faster to invalidly cued trials (VALIDITY, *F*(1,15) = 13.09, *p*<.003). In the 25% condition the ANOVA indicated faster reactions to S_2_ appearing at the right side of the screen (STIMULUS SIDE, *F*(1,15) = 6.20, *p* = .03) but no interactions with this effect were found. If the individual validity effect in either the 25% or the 75% validity condition deviated by more than one standard deviation from the group mean into the opposite direction, this subject's data were excluded from the group EEG time-frequency analysis. Therefore, subjects 3 and 10 were excluded from the analysis.

#### Time-frequency EEG

##### Occipital alpha-rhythm suppression (8–12 Hz)

In all conditions the occipital alpha-rhythm was suppressed. This suppression began about 300 ms after the S_1_ stimulus, increased after the S_2_ stimulus, and continued even 500 ms after the S_2_ (figure 2d). Whereas in the 75% validity condition the alpha-rhythm was more strongly suppressed contralaterally, suppression was stronger ipsilaterally in the 25% condition (figure 2e, f) (VALIDITY CONDITION×STIMULUS SIDE×ELECTRODE HEMISPHERE interaction effects and Bonferroni corrected post-hoc t-tests in two time windows: 400–500 ms, (F(1,13) = 6.99, p<0.01, t(1,13) = −2.70, p<0.02; 500–600 ms: F(1,13) = 5.76, p<0.01; t(1,13) = −2.72, p<0.02). (Table 3 comprises the occipital alpha-rhythm effects from 500–600 ms.)

**Table 3:** EEG results occipital alpha-rhythm suppression experiment II

Condition	Occipital alpha-rhythm suppression in *db*	
	Mean	Std. error
Contralateral_50	−.85	.23
Ipsilateral_50	−.67	.27
Contralateral_75	−1.18	.29
Ipsilateral_75	−.99	.29
Contralateral_25	−.98	.22
Ipsilateral_25	−1.11	.25

For the three experimental conditions (50%, 75% or 25% cue validity), the mean occipital alpha-rhythm suppression (including standard error of the mean) in the time window from 500–600 ms is given.

**Figure 5:**
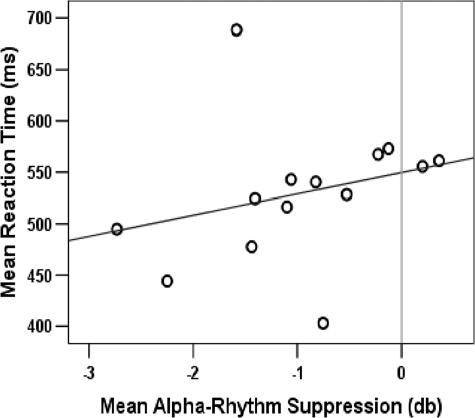
Correlation between reaction times and alpha-rhythm suppression. The scatter plot illustrates the positive inter-subject correlation (r = .50, p<.04, N = 14) between the mean alpha-rhythm suppression in the ISI and the mean reaction time to the upcoming visual targets. Black dots represent the individual subjects' means across the three validity conditions.

As illustrated in figure 5 the mean occipital alpha-rhythm amplitude (across all validity conditions) in the ISI was correlated with the mean RT (r = .50, p<0.04, one-tailed, nonparametric correlation). In other words, subjects with a more strongly suppressed alpha-rhythm tended to respond faster.

##### Central mµ-rhythm suppression (8–12 Hz)

In all conditions, the central mµ-rhythm was suppressed in the ISI beginning about 300 ms after the S_1_ stimulus as well as following the S_2_ beginning about 300 ms after the stimulus and still persisting 500 ms after the S_2_ stimulus (figure 3c). Suppression was significantly stronger in both the 75% and the 25% than in the 50% validity condition (VALIDITY CONDITION effects and Bonferroni corrected post-hoc tests in two time windows: 500–600 ms: F(1,13) = 7.05, p<0.01, 50% versus 75%: p<0.01; 600–700 ms: F(1,13) = 7.30, p<0.01, 50% versus 75%: p<0.02, 50% versus 25%: p<0.04) (figure 3c, d).

##### Central beta-rhythm suppression and rebound (15–22 Hz)

The central beta-rhythm was suppressed between about 200–500 ms after the S_1_ stimulus (figure 3c, e), followed by a rebound within the 500–800 ms time range contralaterally to the S_1_ stimulus (figure 3c, f).

##### Prefrontal gamma-rhythm enhancement (25–35 Hz)

Gamma-activity at prefrontal sites increased immediately preceding the target presentation (600–700 ms) (figure 4c, d); this increase was stronger for cues to the right index finger (STIMULUS SIDE, F(1,15) = 10.30, p<0.007). Although the effect was pronounced in the 50% validity condition there was no significant difference between the three conditions.

#### Main EEG results-experiment II

This experiment aimed to dissociate effects of endogenous versus exogenous attention using an identical crossmodal cueing task. The validity of the tactile cues was set at either 50%, 75%, or 25% validity and subjects were instructed to shift their attention accordingly. As in experiment I, the occipital alpha-rhythm was suppressed in the ISI between S_1_ and S_2_. Interestingly, despite the identical tactile cue, the suppression was stronger contralaterally in the 75% condition and was inverted in the 25% validity condition. Importantly, this lateralization did not depend on the exogenously attended side but on the focus of endogenous attention. Furthermore, faster reactions were related to a stronger occipital alpha suppression.

## Discussion

The goal of this study was to explore the mechanisms underlying crossmodal spatial attention shifts preceding the presentation of a target. In the first experiment, we studied the influence of spatial attention on ongoing oscillatory brain activity in the ISI between a tactile cue and a visual target. In the second experiment we modified cue validity stepwise between 25% and 75%, to dissociate effects of exogenous versus endogenous attention shifts. The spatial attention task showed robust behavioral validity effects and had three effects on EEG-activity: First, it suppressed the occipital alpha-rhythm; this suppression was stronger contralaterally to the attended side and depended on endogenous attention. The amount of alpha-rhythm suppression allowed to predict response speed to the upcoming target. Second, the lateralization of the sensorimotor beta-rhythm rebound was markedly stronger in the cueing task than in the preceding control task. Third, the prefrontal gamma-band activity was enhanced. Thus, we measured cue-related modulations of cortical rhythms preceding the task relevant stimulus. We argue that the effects in the alpha and the gamma-band may represent mechanisms accompanying crossmodal spatial attention, expectation or decision, and therefore might contribute to cue validity effects to the target stimuli.

In agreement with previous spatial cueing studies that investigated endogenous attention shifts induced by visual or auditory cues [39], [40], [43], [44], we found a suppression of alpha-rhythm oscillations in the ISI following tactile cues. In spatial attention paradigms, the alpha-rhythm is often lateralized with either an alpha suppression contralateral to the attended side [37], [39], [40], [43], [44] or an alpha increase contralateral to the ignored side [41], [42]. The suppression of alpha oscillations has been interpreted as a correlate of cortical activation [e.g. 49]–[51] indicating a release of functional inhibition [52]–[54]. An EEG-study by Hummel and coworkers provides further support for the hypothesis that alpha synchronization represents active inhibition since the power of 11–13 Hz oscillations increased over sensorimotor areas when subjects had to inhibit a trained motor response [55]. Similarly, the parieto-occipital alpha amplitude increased during periods of neglecting input from a modality [56], [57]. The alpha suppression following the tactile cues in our study was more pronounced on the side contralateral to the expected target stimulus side, while no suppression occurred in the control tasks. Both findings support the hypothesis of an alpha suppression related to visuo-spatial attention regardless of cue modality.

In our second experiment, we aimed to dissociate effects of exogenous versus endogenous attention by manipulating the cue validity between 25% and 75%. The lateralized tactile cue stimuli induced a transient *exogenous* shift of attention, which was, however accompanied by an *endogenous* shift of attention only in the 75% and in the 25% cue validity conditions. Since exogenous and endogenous attention were shifted to opposite sides specifically in the 25% condition, this condition induced a ‘conflict’ between exogenous and endogenous attention. As to be expected, the lateralization of the alpha suppression in the ISI inverted in the 25% cue validity condition in line with the focus of endogenous attention, here towards the side of the cue. The alpha suppression was not lateralized depending on the side of the stimulation and corresponding to the focus of exogenous attention, i.e. the side of the cue. Furthermore, a stronger alpha suppression during the ISI correlated with the behavioral performance of the subject, indicating faster reactions. Thus, a stronger alpha suppression in this task signals a better preparation for perceiving a visual target and reacting to it. This between-subjects effect was small but significant. It was not present for the mean alpha-suppression in the ISI of the cueing task in experiment I, which is probably due to a smaller number of trials and hence lower statistical power.

Somatosensory stimulation is followed by a suppression and then a rebound of beta frequency oscillations at central scalp locations; both, median nerve stimulation [58]–[60] and tactile stimulation induce these effects [61]. The beta rebound is suppressed by motor cortex excitation related to active exploration of an object, to finger movements, passive motor stimulation, and even to motor imagery [58]–[60], [62]. Computational modeling of the human sensorimotor beta-rhythm using a Hodgkin-Huxley model indicates that the rebound is due to the gamma-aminobutyric acid (GABA)-ergic inhibition of inhibitory interneurons [63]. Surprisingly, in our study the beta rebound was more strongly lateralized in the cueing task than in the control tasks. This indicates that the sensorimotor beta rebound is influenced by the amount of task relevance of the somatosensory stimuli.

We observed a slight but significant enhancement of prefrontal gamma activity (25–35 Hz) specifically in the cueing task during the ISI. The prefrontal cortex has been implicated in a range of functions of higher order cognitive control and integration. We can only speculate about the prefrontal gamma increase we found immediately before target presentation. This increase might represent (I) allocation of spatial attention to the cued side [64], (II) short term memory processing of the cue position [65], (III) response preparation [66], (IV) temporal integration [67], (V) expectation of the task relevant visual stimulus [68], [69], and (VI), aspects of the decision process (“Is the square in the upper or lower half of the screen?”) [70], [71]. In any case, we would argue that the gamma-rhythm enhancement in our experiments is unlikely to be due to eye activity because of a different topography of the effect at the Fp-electrodes than at the upper eye electrode.

In summary, we conclude that crossmodal spatial attention not only influences the evoked brain activity after stimulus onset but also modulates the ongoing brain activity before stimulus onset. Our study describes induced modulations in three frequency bands during a period preceding a target. In line with previous studies, the lateralized alpha suppression to tactile cues indicates endogenous rather than exogenous shifts of visuo-spatial attention independent of cue modality. The correlation between alpha suppression and reaction time supports the hypothesis that alpha suppression indicates cortical activation leading to a higher preparedness to process and to react to the upcoming target. The prefrontal gamma-rhythm enhancement immediately preceding the target might be related to spatial attention, short term memory processing, response preparation, expectation, or decision making. The beta rebound modulation can be regarded as an effect of an enhanced processing of task relevant tactile stimuli. The dynamics of oscillations in the alpha and the gamma-band likely represent mechanisms accompanying crossmodal spatial attention, expectation or decision making, and thereby contribute to cue validity effects to the target stimuli.

## Supporting Information

Figure S1 Grand average of time-frequency modulations at the upper eye electrode in experiment I. The amount of stimulus-locked oscillations at various times and frequencies in relation to the pre-stimulus baseline is color-coded and is expressed in db. Positive values indicate increases, negative values indicate decreases. Zero on the x-axis corresponds to the onset of the S1 stimulus; the vertical black line marks the onset of the S2. The gamma-rhythm enhancement at the eye electrode appears to be later in time and higher in frequency than the gamma-rhythm enhancement reported for the Fp-electrodes.
(9.28 MB TIF)
