# Supplementary figures and images for: What Happens in Between? Human Oscillatory Brain Activity Related to Crossmodal Spatial Cueing
Source: PLoS One. 2008 Jan 23;3(1):e1467. doi: 10.1371/journal.pone.0001467 (PMC2186384; doi:10.1371/journal.pone.0001467)

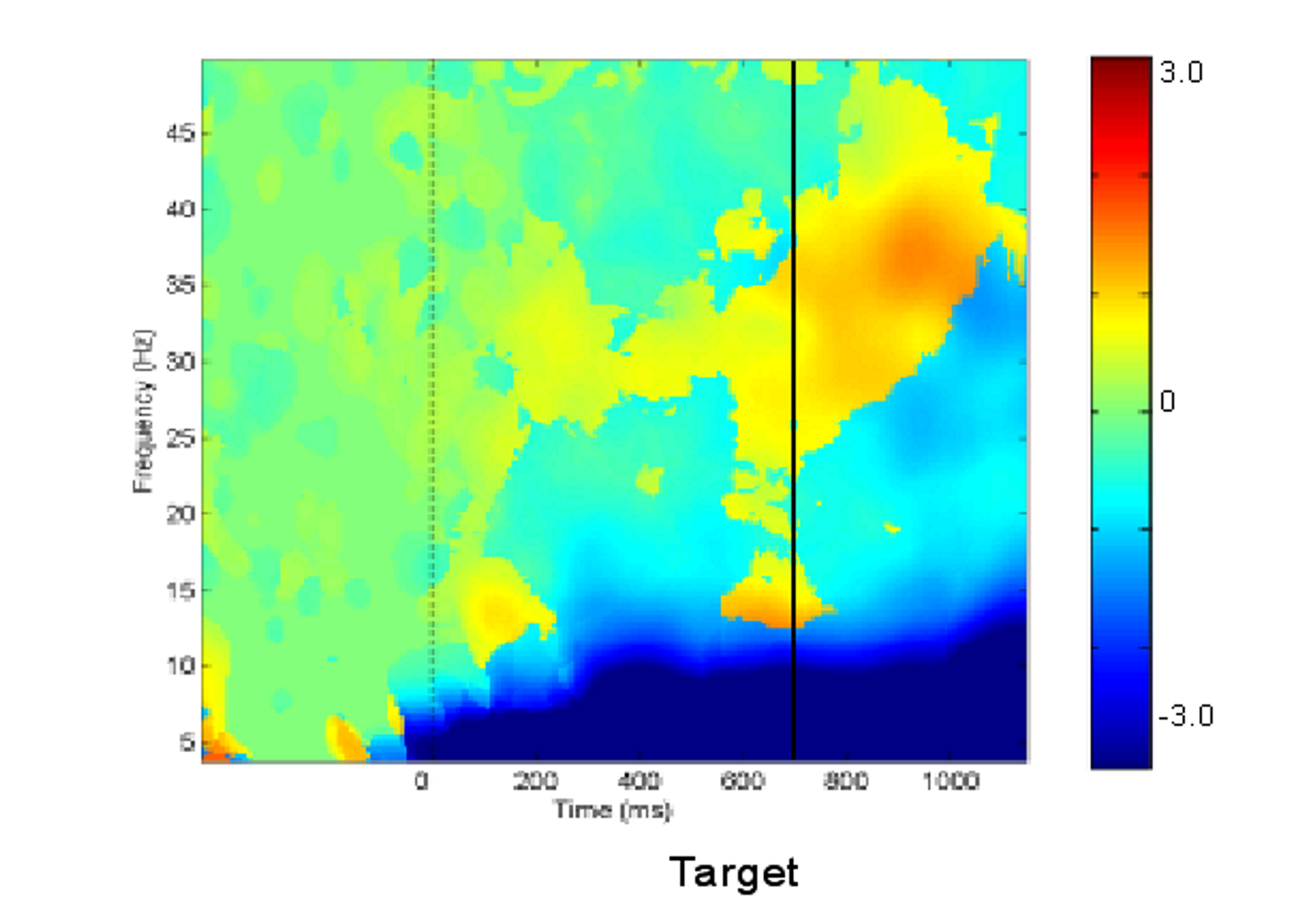

Supplement: Figure S1 — Grand average of time-frequency modulations at the upper eye electrode in experiment I. The amount of stimulus-locked oscillations at various times and frequencies in relation to the pre-stimulus baseline is color-coded and is expressed in db. Positive values indicate increases, negative values indicate decreases. Zero on the x-axis corresponds to the onset of the S1 stimulus; the vertical black line marks the onset of the S2. The gamma-rhythm enhancement at the eye electrode appears to be later in time and higher in frequency than the gamma-rhythm enhancement reported for the Fp-electrodes. (9.28 MB TIF) [file pone.0001467.s001.tif]
